# Circulating miR-122 Is a Predictor for Virological Response in CHB Patients With High Viral Load Treated With Nucleos(t)ide Analogs

**DOI:** 10.3389/fgene.2019.00243

**Published:** 2019-03-22

**Authors:** Yin Wu, Chang Gao, Shaohang Cai, Muye Xia, Guichan Liao, Xiaoyong Zhang, Jie Peng

**Affiliations:** ^1^State Key Laboratory of Organ Failure Research, Guangdong Provincial Key Laboratory of Viral Hepatitis Research, Department of Infectious Diseases, Nanfang Hospital, Southern Medical University, Guangzhou, China; ^2^Department of Pathology, Sun Yat-sen University Cancer Center, Guangzhou, China

**Keywords:** microRNA-122, chronic hepatitis B, virological response, high viral load, nucleos(t)ide analogs

## Abstract

Chronic hepatitis B (CHB) infection remains worldwide health problem. Antiviral treatment options for CHB patients include nucleos(t)ide analogs (NAs) and interferon. Most of the current biomarkers for predicting treatment response are virus-dependent. MicroRNA-122 is the most abundant liver-specific miRNA and has been identified involved in multiple liver physiology and pathology including hepatotropic virus infection. To identify the role of miR-122 in NA therapy, 80 CHB patients with high viral load (HVL) were enrolled and serum miR-122 levels at baseline, week 12 and week 24 were measured. Serum miR-122 levels were significantly lower in patients who developed virological response (VR), compared with non-VR group. Levels of miR-122 at week 12 and week 24 were determined to be independent prognostic indicators for a VR with satisfactory AUROC values at 0.812 and 0.800, respectively. During NA therapy, serum miR-122 level deceased steadily and an earlier reduction was observed in VR group, indicating early reduction of miR-122 level might increase the possibility of developing virological response. In conclusion, we identified the dynamic change of serum miR-122 level and miR-122 levels at week 12 and week 24 as independent predictors for VR in CHB patients with HVL treated with NAs.

## Introduction

Over 240 million people worldwide are living with chronic hepatitis B virus (HBV) infection, and China is one of the countries with the highest population of HBsAg-positive individuals (Schweitzer et al., 2015). Chronic HBV infection can lead to fatal complications including liver cirrhosis, liver failure, and hepatocellular carcinoma (HCC), resulted from repeated flares and continuous inflammation (Chang and Lewin, 2007).

The clinical outcome of infection depends on a complex interaction between viral factors and host immune response (Dandri and Locarnini, 2012). Currently, antiviral treatment options for chronic hepatitis B (CHB) patients include nucleos(t)ide analogs (NAs) and interferon (Lampertico et al., 2017). Previous studies have identified several predictors for treatment response to NA therapy, such as alanine aminotransferase (ALT) (Perrillo et al., 2002; Wong et al., 2018), interferon-inducible protein 10 (Jaroszewicz et al., 2011; Wong et al., 2016), hepatitis B surface antigen (Jaroszewicz et al., 2011; Chen et al., 2014; Wang et al., 2014) and hepatitis B core antibody levels (Fan et al., 2016; Cai et al., 2018). Yet, most of these predictors are virus-dependent, moreover, only a handful are performed in CHB patients with high viral load (HVL) at baseline.

MicroRNAs (miRNAs) are a large class of small non-coding RNAs that post-transcriptionally regulate one third of human genes. These approximately 22-nucleotides long RNAs play important roles in physiological processes as well as occurrence and development of human diseases. Likewise, they exhibit the potential as disease biomarkers and therapeutic targets (Hammond, 2015). MiRNAs, one of the host factors influencing virus-host interactions, play an important role on regulation of HBV replication and host cell metabolism (Deng and Lu, 2016). A large quantity of miRNAs are involved in control of HBV replication. For instance, miR-26b was identified to decrease HBV enhancer/promoter activities and inhibit viral transcription (Zhao et al., 2014), miR-199a-3p and miR-210 show the ability to reduce HBsAg expression (Zhang et al., 2010), miR-125a-5p interferes with the viral sequence (Potenza et al., 2011) and miR-204 was found to target HBV mRNAs (Huang et al., 2016).

In mammals, miR-122 is the most abundant liver-specific miRNA, almost undetectable in other tissues (Lagos-Quintana et al., 2002; Landgraf et al., 2007), and involved in multiple liver physiology and pathology including liver development, lipid metabolism, hepatotropic virus infection and development of HCC (Hu et al., 2012). *In vitro* studies have reported that downregulation of endogenous miR-122 enhanced HBV replication, whereas upregulated miR-122 led to suppression of HBV (Wang et al., 2012). Furthermore, circulating miR-122 in CHB patients was identified to positively correlate with ALT and HBV DNA levels (Waidmann et al., 2012; Xing et al., 2014; Akamatsu et al., 2015), implying that circulating miR-122 levels have the potential to reflect liver damage and the magnitude of viral infection, and might further indicate the outcomes of antiviral therapy.

The aim of this study was to identify the role of serum miR-122 levels as predictors for treatment response in CHB patients with pre-treatment HVL treated with NAs. Secondary, we aim to explore the dynamic changes of miR-122 during NA treatment.

## Materials and Methods

### Study Subjects

This study was comprised of 80 CHB patients with HVL (HBV DNA >10^7^ copies/mL) at baseline, enrolled in a prospective, multicenter, controlled trial, treated with lamivudine (LAM) and adefovir (ADV) (*n* = 38) or entecavir (ETV) (*n* = 42). All patients provided written informed consent and received NA therapy for up to 96 weeks. The trial has been approved by the Institutional Review Board of Nanfang Hospital, Southern Medical University (ID: ZHF2011206), and described in detail previously (Cai et al., 2016, 2018). In addition, patients who did not have stored serum samples at week 0, 12, and 24 during the follow-up treatment, were excluded from this study. Treatment response was determined after 1-year (week 48) and 2-year (week 96) treatment. A virological response (VR) was defined as HBV DNA levels <300 copies/mL.

### Serological Methods

Serum HBsAg, HBeAg, HBeAb levels were measured by the ARCHITECT i2000SR system (Abbott Laboratories, United States). Serum HBV DNA levels were measured by the Cobas Ampliprep and Cobas TaqMan, version 2.0 (CAP/CTM, Switzerland), with a detection limit ranging from 20 to 1.7 × 10^8^ IU/mL (1 IU/mL = 5.82 copies/mL).

### MicroRNA Isolation and Quantitative Real-Time PCR

The isolation of serum miRNA-122 was performed by the miRNeay Serum/Plasma Kit (QIAGEN, Germany) according to the manufacturer’s instructions and *Caenorhabditis elegans* miR-39 was used to act as an internal control. The cDNA was synthesized by miScript Reverse Transcription Kit (QIAGEN) and quantification of miRNA was performed with miScript SYBR Green PCR Kit (QIAGEN) by using LightCycler 480 (Roche Diagnostics, Switzerland). The results were normalized against 10^6^ copies of *C. elegans* miR-39 miRNA transcripts, same as our previous study (Ge et al., 2017), and presented as the log_10_ normalized value for appropriate display, shown as lg miR-122/10^6^ miR-39 copies.

### Statistical Analysis

Continuous variables were shown as mean ± SD or median as appropriate. Differences between two groups were analyzed by Mann-Whitney *U*-test and Chi-squared test, when applicable. Dynamic changes in serum miR-122 level were evaluated by repeated measures analysis. Receiver operating characteristic (ROC) curves were used to determine the accuracy of using serum miR-122 levels to predict treatment response to NA therapy. All of the statistical analyses were performed with SPSS Statistics 24.0 and GraphPad Prism 7.0. *P*-values of <0.05 were considered statistically significant.

## Results

### Patient Characteristics

Baseline characteristics of all patients enrolled in this study are shown in Table 1. All patients were HBeAg-positive. At week 48 and 96, 41% (33/80), and 84% (67/80) patients achieved virological response, respectively. The pre-treatment serum miR-122 levels in patients treated with combination therapy (LAM + ADV) and ETV monotherapy were 84364 ± 123355/10^6^ miR-39 copies and 119081 ± 367141/10^6^ miR-39 copies, respectively. There were no significant differences between the two groups in distribution of both demographics and clinical characteristics at baseline.

**Table 1:** Patient characteristics.

		LAM+ADV group (*n* = 38)	ETV group (*n* = 42)	*p*-value
Age (years)		29.9 ± 8.5	29.9 ± 8.1	0.912
Gender, male (%)		28 (73.7)	33 (78.6)	0.608
ALT (U/L)		229.5 ± 137.0	246.3 ± 162.7	0.877
HBV DNA (log_10_IU/mL)		7.95 ± 0.57	7.99 ± 0.69	0.345
miR-122/10^6^ miR-39 copies		84364 ± 123355	119081 ± 367141	0.977
Virological response	week 48	14 (36.8)	19 (45.2)	0.446
	week 96	33 (86.8)	34 (81.0)	0.476


*Continuous variables are shown as mean ± SD, categorical variables as n (%)*.

### Serum miR-122 Levels Positively Associated With Serum HBV DNA Levels in CHB Patients

**FIGURE 1:**
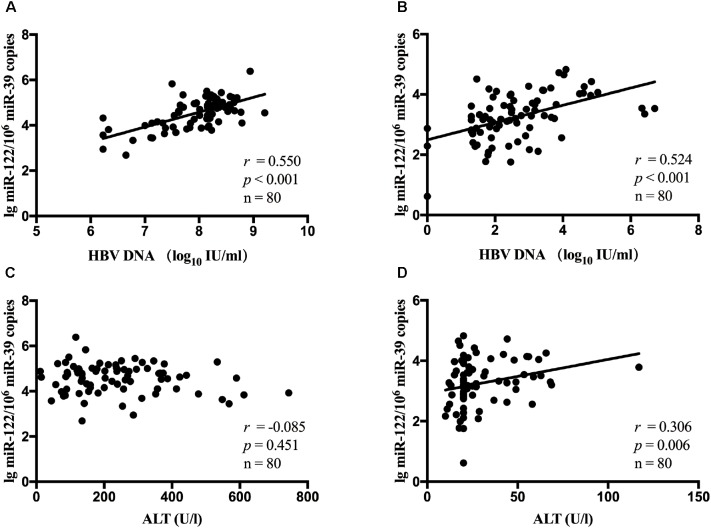
The correlation between serum miR-122 levels, viral loads and ALT levels in all patients. Spearman’s correlations of serum miR-122 levels and HBV DNA levels were conducted at baseline **(A)** and week 24 **(B)**. Spearman’s correlations of serum miR-122 levels and ALT levels were conducted at baseline **(C)** and week 24 **(D)**.

It has been estimated that miR-122 played a role in chronic HBV infection. Hence, in all patients, the association between serum miR-122 levels, viral loads and ALT levels was evaluated. A significant correlation between miR-122 levels and viral loads was observed at baseline (*r* = 0.550, *p* < 0.001, Figure 1A) and week 24 (*r* = 0.524, *p* < 0.001, Figure 1B), using spearman analysis. However, no correlation was found between miR-122 levels and ALT levels at baseline (*r* = -0.085, *p* = 0.451, Figure 1C), whereas at week 24, a significant correlation was observed (*r* = 0.306, *p* = 0.006, Figure 1D).

### Serum miR-122 Levels Discriminated Different Patient Groups of Treatment Response

**FIGURE 2:**
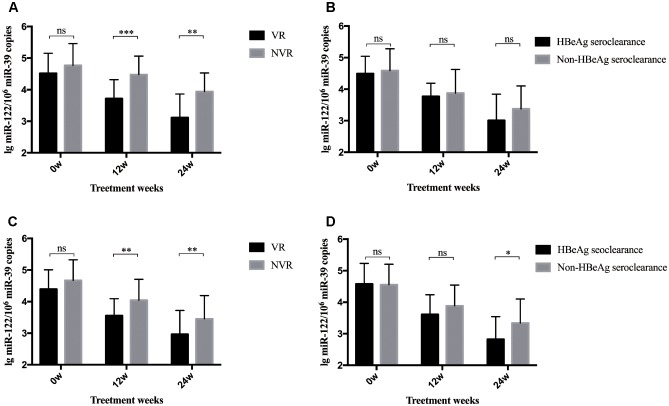
Differential levels of serum miR-122 among subgroups. The bars represent the mean and standard error of mean. **(A)** MiR-122 levels in serum at baseline, week 12, and week 24 among patient groups whether a VR was achieved at week 96. **(B)** MiR-122 levels at baseline, week 12, and week 24 among patient groups whether HBeAg clearance was achieved at week 96. **(C)** MiR-122 levels in serum at baseline, week 12, and week 24 among patient groups whether a VR was achieved at week 48. **(D)** MiR-122 levels at baseline, week 12, and week 24 among patient groups whether HBeAg clearance was achieved at week 48. VR, virological response; NVR, non-virological response; ns, not significant; ^∗^*p* < 0.05; ^∗∗^*p* < 0.01; ^∗∗∗^*p* < 0.001.

A total of 80 patients were divided into two groups according to whether they achieved a VR after 96-week NA treatment. As shown in Figure 2A, patients with a VR at week 96 showed significantly lower serum miR-122 levels at week 12 (*p* < 0.001) and 24 (*p* = 0.001) than those without a VR at week 96. Yet, these two subgroups showed approximate levels of miR-122 at baseline. Given the results and the positive correlation between miR-122 and viral loads, higher miR-122 levels in serum after 12 to 24-week NA therapy might indicate the suboptimal treatment response and impediment to cure. Similar results were observed among patients with or without a VR at week 48. At baseline, patients in VR and non-virological response (NVR) showed approximate miR-122 levels, whereas at week 12 and 24, significantly lower miR-122 levels were observed in VR group (*p* = 0.002 and *p* = 0.006, respectively) (Figure 2C). Furthermore, patients with HBeAg seroclearance at week 48 showed lower serum miR-122 levels than those with sustained positive HBeAg (*p* < 0.001) (Figure 2D), regretfully, similar differential level was not found between patients whether HBeAg seroclearance was developed at week 96 (Figure 2B).

### The Predictive Value of Serum miR-122 Levels for Virological Response in CHB Patients Treated With NAs

**FIGURE 3:**
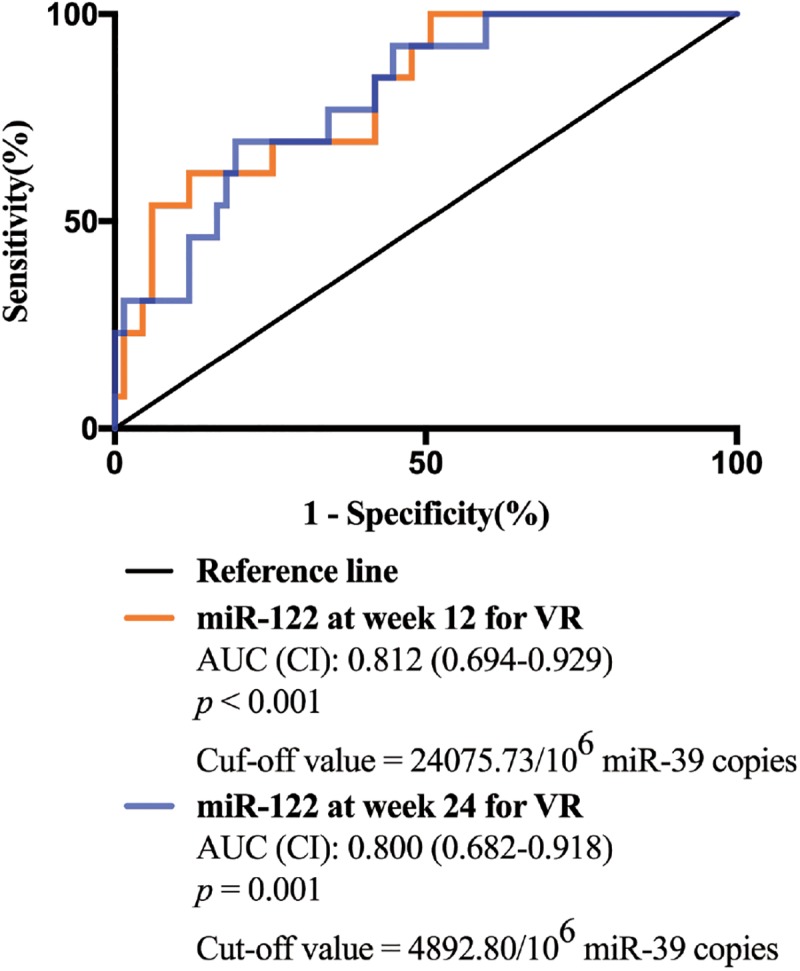
The receiver operating characteristic (ROC) curve analyses of the sensitivity and specificity of miR-122 levels in serum for the prediction of virological response at week 96. Both miR-122 levels at week 12 and week 24 were analyzed.

Univariate and multivariate analyses were performed to identify the associations between serum miR-122 levels and VR at week 96. In univariate analysis, miR-122 levels at week 12 and 24 were significantly associated with a VR at week 96 (*p* = 0.001 and *p* = 0.001, respectively) (Table 2), increased the possibility of VR and served as protective factors in antiviral process. Similar results were revealed in multivariate analysis, indicating miR-122 levels as independent prognostic indicator. Furthermore, to evaluate the predictive value of serum miR-122 levels for VR in CHB patients treated with NAs, the ROC curve was generated. The optimal cut-off value for miR-122 levels in serum at week 12 and 24 were 24075.73/10^6^ miR-39 copies and 4892.80/10^6^ miR-39 copies, respectively. The sensitivity of miR-122 levels at week 12 for prediction of a VR at week 96 was 88.1% with a specificity of 61.5%, the sensitivity of miR-122 levels at week 24 was 80.6% with a specificity of 69.2% (Figure 3). The results suggested that lower miR-122 level in serum at week 12 and 24 might contribute to the immune control and indicate better clinical outcomes in CHB patients treated with NAs.

**Table 2:** Univariate and multivariate analyses of associations between miR-122 levels and virological response at week 96.

Variables	Univariate analysis			Multivariate analysis		
	OR	95% CI	*p-*values	OR	95% CI	*p*-values
miR-122 level at baseline	0.530	0.196–1.436	0.212			
miR-122 level at week 12	0.132	0.041–0.421	***0.001***	0.217	0.058–0.820	***0.024***
miR-122 level at week 24	0.139	0.042–0.462	***0.001***	0.278	0.079–0.983	***0.047***
Age	1.013	0.940–1.092	0.734			
Gender	1.870	0.376–9.299	0.444			
ALT at baseline	1.005	0.999–1.010	0.079			
HBV DNA at baseline	0.264	0.065–1.062	0.061			


*VR, virological response; NVR, non-virological response. *P*-value <0.05 is shown in bold and italics*.

### Dynamic Change of miR-122 Level During NA Therapy

**FIGURE 4:**
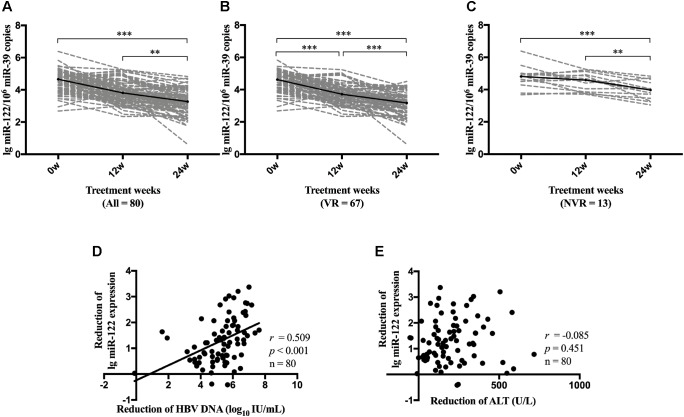
Dynamic change of miR-122 level during NA therapy. Dynamic change of miR-122 levels among all patients **(A)**, VR group **(B)** and NVR **(C)** group were evaluated. Spearman’s correlations between reduction of serum miR-122 levels and the reduction of viral load **(D)** or the reduction of ALT levels **(E)** were conducted at week 24. VR, virological response; NVR, non-virological response. ^∗∗^*p* < 0.01; ^∗∗∗^*p* < 0.001.

We further performed repeated measures analysis to determine the dynamic change of serum miR-122 level during NA therapy. Compared with miR-122 levels at baseline, all patients demonstrated a significant reduction of miR-122 level at week 24 (*p* < 0.001) (Figure 4A). VR patients showed a consistent reduction of miR-122 at both week 12 (*p* < 0.001) and week 24 (*p* < 0.001) (Figure 4B), whereas NVR patients displayed a downward but not significant trend at week 12 (*p* = 0.092) and significant reduction only at week 24 (*p* = 0.001) (Figure 4C). Reduction of miR-122 level at week 24 was observed to be significantly associated with reduction of viral load (*p* < 0.001) (Figure 4D), whereas no significant correlation was found between reduction of miR-122 and reduction of ALT levels at week 24 (Figure 4E).

## Discussion

In this study, we investigated the differential level of miR-122 in CHB patients with different clinical outcomes and the dynamic change of miR-122 level in serum during NA treatment. We found that serum miR-122 levels steadily decreased during antiviral therapy and higher level of miR-122 was associated with suboptimal treatment response. Serum miR-122 level at week 12 and week 24 were identified to be satisfactory predictors for a VR at week 96.

MiR-122 has been recently studied increasingly to identify its role in hepatotropic virus infection. It has been demonstrated that miR-122 level in hepatocytes is down-regulated in CHB patients, and suppressed miR-122 can contribute to viral persistence through modulating inactivation of IFN expression and cyclin G(1)-modulated p53 activity (Wang et al., 2012; Gao et al., 2015), indicating this liver-specific miRNA closely involved in host-mediated antiviral defense. Given the complex methods of detecting miR-122 in liver, circulating miR-122 level displays its potential as a specific biomarker. In HBV-infected patients without any antiviral therapy, lower serum miR-122 levels exhibited a higher spontaneous HBsAg seroclearance rate (Akuta et al., 2018).

Therefore, in this study, we aimed to identify the predictive value of serum miR-122 levels and the dynamic change of miR-122 level during NA therapy in CHB patients. In agreement with previous studies (Waidmann et al., 2012; Xing et al., 2014; Akamatsu et al., 2015), a significant and positive correlation between serum miR-122 level and viral loads was observed. However, serum miR-122 levels showed no association with ALT levels at baseline, which may be on account of the special patient cohort this study performed in. All patients enrolled in this study were with HVL of HBV DNA >10^7^ copies/mL at baseline. A weak T cell response is often exhibited in CHB patients, with depletion of T cells and diminished cytotoxic capacity (Cho et al., 2017). This phenomenon which is called as T cell exhaustion, leads to persistence of chronic HBV infection and reduction of liver damage (Chang and Lewin, 2007). Serum ALT activity, known as an essential biomarker signaling liver damage, might be limited in CHB patients with HVL at baseline. These results indicated that miR-122 is related to viral replication rather than liver damage. Moreover, we observed significantly lower serum miR-122 levels at week 12 and week 24 in patients who developed VR at week 96 compared to non-responders. Circulating miR-122 levels at both week 12 and week 24 displayed independent prognostic value for a VR to NA therapy. In addition, we demonstrated that AUROC values for serum miR-122 were satisfactory at 0.812 and 0.800, a miR-122 level of <24075.73/10^6^ miR-39 copies at week 12 and 4892.80/10^6^ miR-39 copies at week 24 may be applied practically in clinical practice.

We further identified the dynamic change of miR-122 level in serum during NA therapy, which remains unknown to date. During antiviral therapy, serum miR-122 level steadily decreased and displayed a significant reduction at week 24, while in patients who developed VR, miR-122 level decreased more rapidly with a significant reduction at week 12. However, further studies were needed to confirm whether prominent reduction of miR-122 level at early phase will contribute to satisfactory treatment response. In addition, lower serum miR-122 level was observed in patients who developed HBeAg seroclearance at week 48. However, up to week 96, similar results were not observed. One possible explanation was the relatively small sample size. Further studies with larger sample size are necessary to determine the role of miR-122 in facilitating HBV clearance, promoting HBeAg seroclearance and even in assisting immune control after NA cessation.

This study had several limitations. One of the limitations was the absence of the contrast between CHB patients and healthy controls. The other was the lack of liver specimens. As only a few patients accepted liver biopsies, we did not measure the miR-122 expression in liver. Yet, it has been found that, compared with healthy controls, miR-122 expression in liver was significantly down-regulated in CHB patients (Wang et al., 2012), whereas serum miR-122 level was significantly increased (Waidmann et al., 2012; Xing et al., 2014). Thus, we speculated that there might be a negative correlation between miR-122 level in liver and in circulating system of CHB patients due to two possible explanations. One is that liver injury induces the secretion of extracellular vesicles containing exosome-associated miRNAs including miR-122 into the bloodstream (Momen-Heravi et al., 2015). The other is that subviral HBsAg particles carry hepatocellular miRNAs (including miR-122) from hepatocytes to bloodstream (Novellino et al., 2012). Both of them lead to transportation of miR-122 from liver to circulating system. The speculation requires further studies to identify. Yet this is the first study to investigate the dynamic change of miR-122 during antiviral therapy, and the first study to identify the prognostic value of miR-122 in non-neoplastic and non-fibrotic CHB patients for treatment response to NA therapy.

In conclusion, we identified the dynamic change of miR-122 levels in serum during NA therapy. Moreover, in CHB patients with HVL treated with NAs, we demonstrated that serum miR-122 levels at week 12 and week 24 could provide practical application of predicting virological response.

## Author Contributions

JP conceived and designed the research. SC and GL collected the samples. YW, CG, and MX performed the experiments. YW analyzed the data and drafted the manuscript with additional input and suggestions from XZ. All authors reviewed and approved the manuscript.

## Conflict of Interest Statement

The authors declare that the research was conducted in the absence of any commercial or financial relationships that could be construed as a potential conflict of interest.
